# Primary Cutaneous Apocrine Carcinoma of the Scalp Arising in Syringocystadenoma Papilliferum: Report of a Rare Case

**DOI:** 10.7759/cureus.113436

**Published:** 2026-07-27

**Authors:** Mayar Ballaji, Ahmad Alhazmi, Sameer Albouq

**Affiliations:** 1 General Surgery, King Fahad Hospital Madinah, Madinah, SAU

**Keywords:** apocrine mixed tumor, primary apocrine, scalp tumor, sebaceous carcinoma, sebaceous cysts

## Abstract

Primary cutaneous apocrine carcinoma (PCAC) is an exceptionally rare malignant adnexal neoplasm arising from apocrine sweat glands. Scalp involvement is particularly uncommon, especially when occurring in association with syringocystadenoma papilliferum (SCAP), making diagnosis and management challenging. We report the case of a 56-year-old woman with a congenital left occipital scalp lesion that remained stable for decades before demonstrating progressive enlargement over the preceding two years. Clinical examination revealed a solitary exophytic scalp mass without palpable cervical lymphadenopathy. Contrast-enhanced computed tomography (CT) demonstrated a superficial scalp lesion without calvarial or intracranial invasion. Histopathological examination following wide local excision established the diagnosis of primary cutaneous apocrine carcinoma arising in a background of SCAP with an incidental superficial basal cell carcinoma. Although immunohistochemistry was not performed, comprehensive clinicoradiological evaluation, including CT of the neck, chest, abdomen, and pelvis, mammography, breast ultrasonography with core biopsy confirming fibroadenoma, and whole-body bone scintigraphy, excluded an extracutaneous primary malignancy and metastatic disease. Surgical margins were negative, and the patient remains disease-free on follow-up. This case highlights the importance of considering malignant transformation in longstanding scalp lesions demonstrating recent clinical change, the role of comprehensive staging investigations in excluding metastatic disease, and the value of complete surgical excision with long-term surveillance in managing this rare malignancy.

## Introduction

Primary cutaneous apocrine carcinoma (PCAC) is an exceptionally rare malignant adnexal neoplasm arising from apocrine sweat glands, accounting for a very small proportion of primary cutaneous malignancies. It predominantly affects middle-aged and elderly individuals without a clear sex predilection and most commonly occurs in regions rich in apocrine glands, particularly the axilla and anogenital region. Scalp involvement is distinctly uncommon, making the present case an unusual clinical presentation [1,2].

Clinically, PCAC usually presents as a slowly enlarging solitary erythematous or violaceous nodule or plaque with a nonspecific appearance that may mimic benign cutaneous lesions. Although many tumors follow an indolent course, some demonstrate locally aggressive behavior with the potential for regional lymph node involvement and distant metastasis involving the lungs, liver, and bones. Consequently, histopathological examination is essential for establishing the diagnosis, particularly in longstanding lesions that exhibit recent enlargement, ulceration, pain, bleeding, or discharge [1,2].

Primary cutaneous apocrine carcinoma may arise de novo or develop in association with pre-existing benign adnexal lesions, most commonly syringocystadenoma papilliferum (SCAP). Malignant transformation of SCAP is uncommon, with basal cell carcinoma representing the most frequently reported associated malignancy. The occurrence of PCAC arising within SCAP is exceptionally rare, with only isolated cases described in the literature, particularly when involving the scalp [3,4].

Histopathological examination remains the cornerstone of diagnosis. Clinicopathological correlation is essential to distinguish primary cutaneous apocrine carcinoma from metastatic adenocarcinoma, particularly metastatic breast carcinoma, which represents the principal differential diagnosis. Although immunohistochemical studies are frequently helpful in diagnostically challenging cases, characteristic histopathological findings together with comprehensive clinicoradiological evaluation may establish the diagnosis in selected patients while excluding an extracutaneous primary malignancy [3,4].

Because of the rarity of PCAC, standardized management guidelines remain unavailable. Wide local excision with histologically negative margins is considered the treatment of choice, while long-term clinical surveillance is recommended because of the risk of local recurrence and distant metastasis. Herein, we report a rare case of primary cutaneous apocrine carcinoma arising in syringocystadenoma papilliferum of the scalp and describe its clinical presentation, histopathological findings, comprehensive staging investigations, surgical management, and short-term outcome [5].

## Case presentation

A 56-year-old woman presented to the General Surgery Clinic with a firm, slowly enlarging lesion over the left occipital scalp. According to the patient, the lesion had been present as a small swelling since birth and had gradually enlarged over the preceding five years, with more rapid growth during the last two years. She reported mild pain and intermittent bleeding from the lesion but denied purulent discharge, constitutional symptoms, or other skin lesions. Her medical history was otherwise unremarkable, with no family history of malignancy.

Physical examination revealed a solitary pinkish lesion arising from the left occipital scalp measuring approximately 2 × 2 cm clinically, with no active discharge at the time of examination. No cervical, preauricular, or postauricular lymphadenopathy was detected, and no other suspicious lesions were identified on head and neck examination.

Routine laboratory investigations, including a complete blood count (CBC), liver function tests (LFTs), and renal function tests (RFTs), were within normal limits.

Computed tomography was performed because of the lesion’s progressive enlargement, ulceration, and clinical suspicion of malignant transformation. The examination was obtained to evaluate the extent of local disease, exclude calvarial or intracranial involvement, and assist in preoperative planning. Cross-sectional imaging is not routinely indicated for all scalp lesions but is recommended when malignancy or deep tissue invasion is suspected.

**Figure 1:**
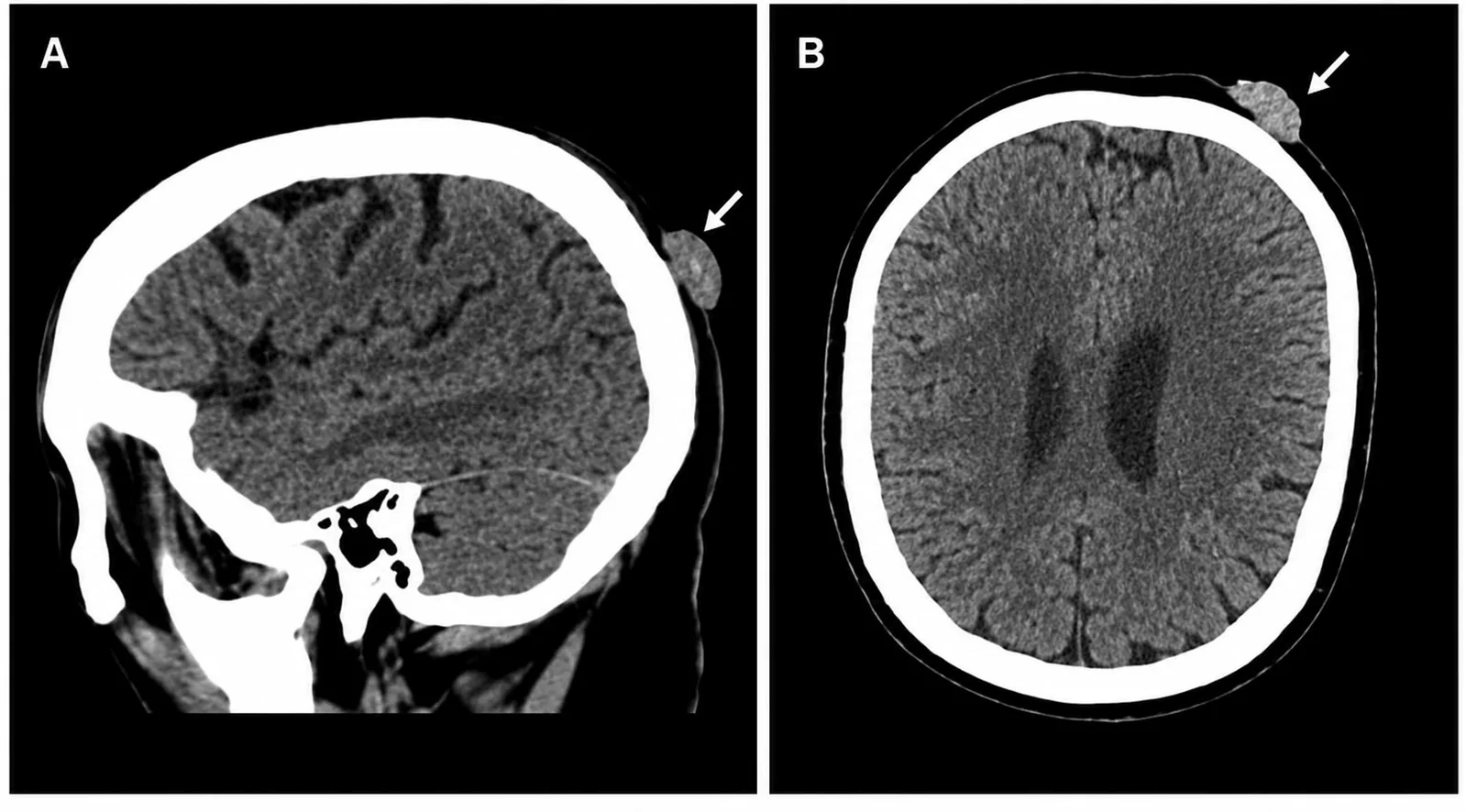
Preoperative computed tomography (CT) of the head demonstrating a scalp lesion without underlying calvarial or intracranial invasion. Sagittal (A) and axial (B) CT images demonstrate a left occipital scalp mass without evidence of calvarial erosion, intracranial extension, or cervical lymphadenopathy.

Contrast-enhanced CT of the head demonstrated a left occipital scalp soft-tissue lesion measuring approximately 2.0 × 1.3 cm with irregular margins and no evidence of calvarial or intracranial extension (Figure 1).

**Figure 2:**
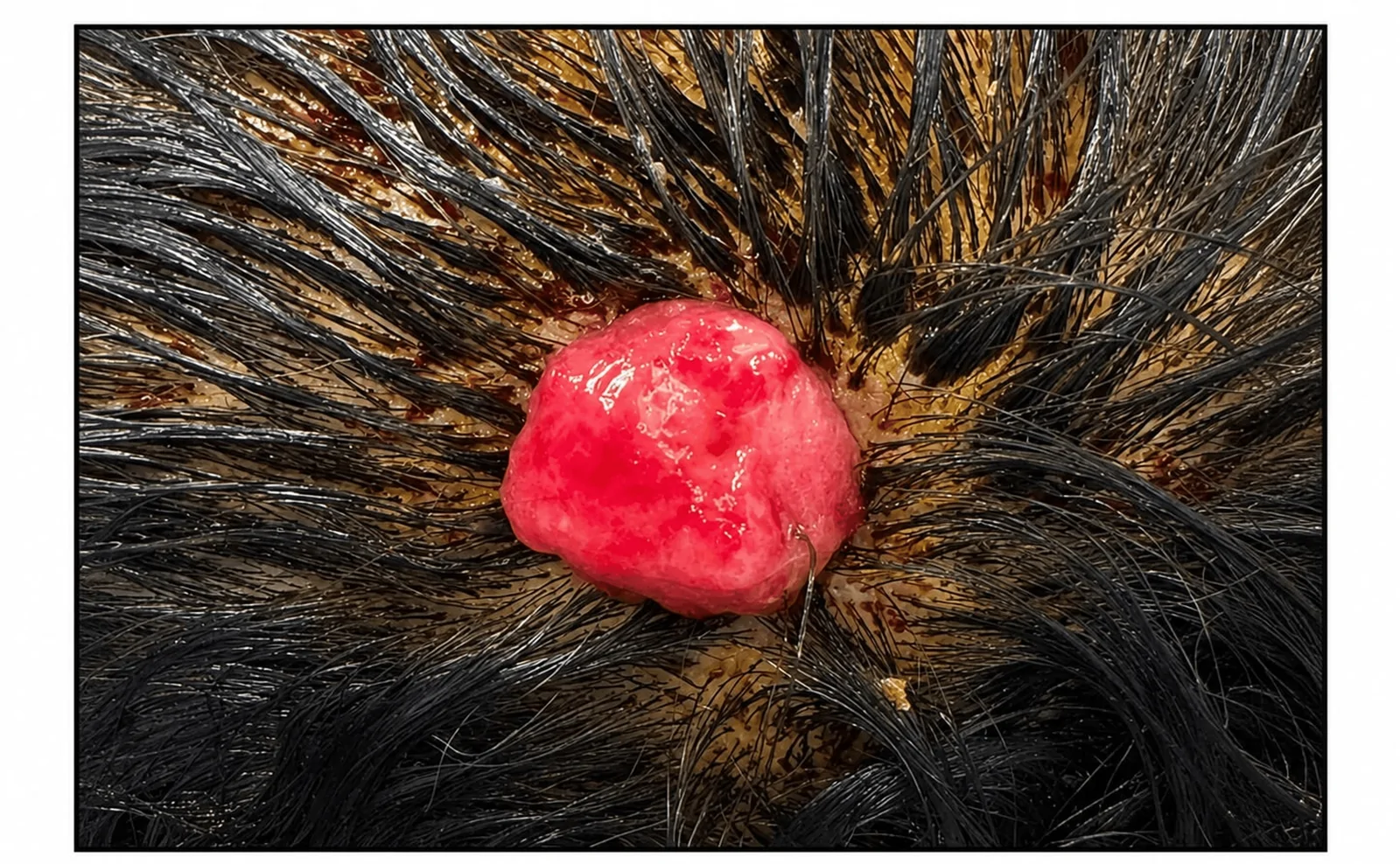
Preoperative clinical appearance of the scalp lesion. Preoperative clinical photograph showing a solitary, well-circumscribed, exophytic erythematous nodule measuring approximately 2.6 cm in greatest diameter, located over the left occipital scalp.

The patient subsequently underwent elective wide local excision of the lesion under local anesthesia. An elliptical incision was made around the lesion, and the specimen was completely excised and submitted for histopathological examination (Figure 2).

**Figure 3:**
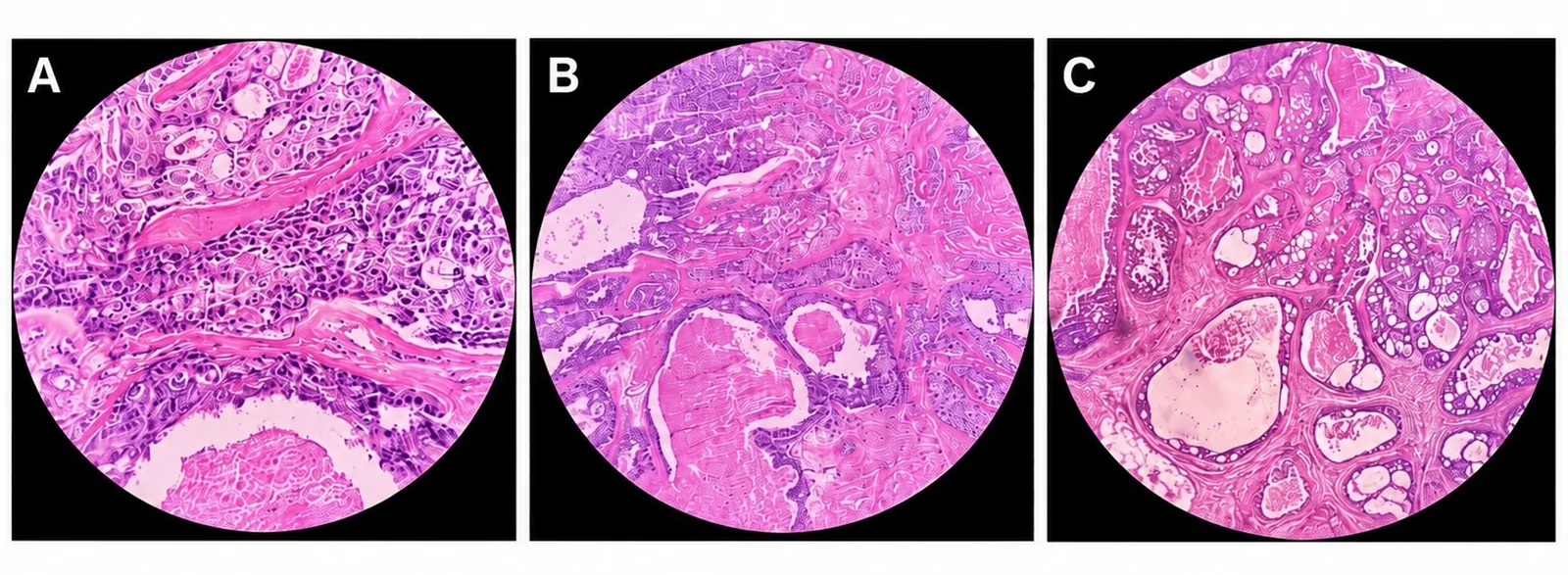
Histopathological features of primary cutaneous apocrine adenocarcinoma arising in syringocystadenoma papilliferum. Representative hematoxylin and eosin (H&E)-stained sections showing (A) a high-power view of malignant apocrine epithelial cells with pleomorphic nuclei and abundant eosinophilic cytoplasm; (B) a low-power view demonstrating the transition from syringocystadenoma papilliferum to invasive apocrine adenocarcinoma; and (C) a low-power view demonstrating the characteristic glandular and cystic architecture of syringocystadenoma papilliferum adjacent to the malignant component.

Histopathological examination demonstrated primary cutaneous apocrine adenocarcinoma arising in the background of syringocystadenoma papilliferum (SCAP). The tumor measured 2.6 cm in greatest dimension and was located 0.4 cm from the closest peripheral skin margin and 0.5 cm from the deep margin. An incidental superficial basal cell carcinoma measuring 0.2 cm in greatest dimension with a thickness of 0.1 cm (Clark level II) was also identified. The basal cell carcinoma was located 0.3 cm from the skin margin and 0.8 cm from the deep margin (Figure 3).

Given the clinical suspicion of malignant transformation, comprehensive staging investigations were performed following multidisciplinary tumor board discussion. Contrast-enhanced CT of the chest demonstrated no evidence of thoracic metastases, mediastinal or hilar lymphadenopathy, or osseous lesions; however, an incidental enhancing lesion was identified in the left breast. CT of the abdomen and pelvis showed no intra-abdominal metastatic disease. High-resolution contrast-enhanced CT of the neck revealed no cervical lymphadenopathy but demonstrated tiny indeterminate lytic foci within the cervical vertebral bodies, prompting further evaluation with a whole-body bone scan. Bone scintigraphy showed no evidence of osseous metastasis. Mammography and breast ultrasonography identified a 1.5 × 0.8 × 0.9 cm circumscribed left breast mass (BI-RADS (Breast Imaging Reporting and Data System) 4A), and ultrasound-guided core biopsy confirmed a benign fibroadenoma. The patient continues regular follow-up in the general surgery clinic with no evidence of local recurrence or distant metastatic disease.

## Discussion

Primary cutaneous apocrine carcinoma (PCAC) is an exceptionally rare malignant adnexal neoplasm arising from apocrine sweat glands. It predominantly affects middle-aged and elderly patients and most frequently occurs in regions rich in apocrine glands, particularly the axilla and anogenital region. Scalp involvement is uncommon, making the present case an unusual clinical presentation. Owing to its rarity and nonspecific clinical appearance, PCAC is frequently mistaken for a benign cutaneous lesion, potentially delaying diagnosis and definitive treatment [1,2].

Primary cutaneous apocrine carcinoma may arise de novo or develop in association with pre-existing benign adnexal lesions, most commonly syringocystadenoma papilliferum (SCAP). Although malignant transformation of SCAP is uncommon, basal cell carcinoma is the malignancy most frequently reported in association with this lesion. The coexistence of primary cutaneous apocrine carcinoma arising within SCAP together with an incidental superficial basal cell carcinoma, as observed in our patient, represents an exceptionally rare pathological finding that has been described only in isolated case reports [3,4].

Clinically, PCAC usually presents as a slowly enlarging solitary nodule or plaque that may become ulcerated or symptomatic with pain, bleeding, or discharge. In our patient, the lesion had reportedly been present since birth as a small scalp swelling and enlarged gradually over several years, with more rapid growth during the preceding two years accompanied by intermittent bleeding. These recent clinical changes raised suspicion for malignant transformation and prompted further diagnostic evaluation. This prolonged clinical course emphasizes the importance of maintaining a high index of suspicion for malignant transformation in longstanding scalp lesions demonstrating recent enlargement or changes in symptoms [2,5].

Histopathological examination remains the gold standard for diagnosis. Although immunohistochemical staining is frequently recommended to distinguish primary cutaneous apocrine carcinoma from metastatic adenocarcinoma, particularly metastatic breast carcinoma, characteristic histopathological findings combined with careful clinicoradiological correlation may establish the diagnosis in selected cases. In our patient, immunohistochemistry and periodic acid-Schiff (PAS) staining were not performed. Nevertheless, the diagnosis was supported by the characteristic histomorphological features of apocrine adenocarcinoma arising in a background of SCAP together with comprehensive radiological assessment that excluded an extracutaneous primary malignancy [1,4,6].

Because metastatic adenocarcinoma, particularly from the breast, represents the principal differential diagnosis, comprehensive staging investigations were undertaken following multidisciplinary tumor board discussion. Contrast-enhanced CT of the neck, chest, abdomen, and pelvis demonstrated no evidence of regional lymphadenopathy or distant metastatic disease. CT of the chest incidentally identified a left breast enhancing lesion, prompting dedicated mammography and breast ultrasonography, which demonstrated a BI-RADS 4A lesion measuring 1.5 × 0.8 × 0.9 cm. Ultrasound-guided core needle biopsy subsequently confirmed a benign fibroadenoma. CT of the neck demonstrated no cervical lymphadenopathy but identified tiny indeterminate lytic foci within the cervical vertebral bodies; therefore, whole-body bone scintigraphy was performed and demonstrated no evidence of osseous metastasis. Collectively, these investigations excluded an alternative primary malignancy and supported the diagnosis of primary cutaneous apocrine carcinoma [4,6].

Because of the rarity of PCAC, there are currently no standardized treatment guidelines. Wide local excision with histologically negative margins remains the treatment of choice and has consistently been associated with favorable local disease control. Regional lymph node dissection is generally reserved for patients with clinically or radiologically suspicious nodal disease, whereas the role of adjuvant radiotherapy or systemic therapy remains uncertain and should be individualized according to pathological risk factors and disease extent [5,7].

The biological behavior of PCAC is variable, ranging from indolent disease to locally aggressive tumors with the potential for regional or distant metastasis. Consequently, prolonged clinical surveillance is recommended even after complete surgical excision. In the present case, complete surgical excision with negative margins was achieved, comprehensive staging demonstrated no evidence of metastatic disease, and the patient remains under regular outpatient follow-up without clinical or radiological evidence of local recurrence or distant metastasis [2,5,7].

A limitation of this report is that immunohistochemical and PAS staining were not performed. However, the diagnosis was supported by the characteristic histopathological findings together with comprehensive clinicoradiological evaluation, including CT of the neck, chest, abdomen, and pelvis, breast imaging with ultrasound-guided core biopsy, and whole-body bone scintigraphy, which excluded an extracutaneous primary malignancy.

This case contributes to the limited literature describing primary cutaneous apocrine carcinoma arising in syringocystadenoma papilliferum of the scalp. It highlights the importance of recognizing recent changes in longstanding scalp lesions, performing appropriate staging investigations to exclude metastatic disease, and managing these patients through multidisciplinary evaluation with complete surgical excision and long-term surveillance [6,8].

## Conclusions

Primary cutaneous apocrine carcinoma arising in syringocystadenoma papilliferum of the scalp is an exceptionally rare adnexal malignancy that may clinically mimic a benign longstanding scalp lesion. Recent enlargement or changes in symptoms should raise suspicion for malignant transformation and prompt histopathological evaluation. Comprehensive clinicoradiological assessment is essential to exclude metastatic disease or another primary malignancy before establishing the diagnosis. Wide local excision with histologically negative margins remains the cornerstone of treatment, while multidisciplinary evaluation and long-term surveillance are recommended because of the potential risk of recurrence and metastasis. Reporting additional well-documented cases will contribute to a better understanding of the clinical behavior, optimal management, and prognosis of this rare neoplasm.
